# Screening mammography in women aged 40–49: Is it time to change?

**DOI:** 10.1186/1477-7800-3-4

**Published:** 2006-02-06

**Authors:** S Helme, N Perry, K Mokbel

**Affiliations:** 1 St. George's Hospital, London, UK; 2 The Princess Grace Hospital, London, UK; 3 St. Bartholomew's Hospital, London, UK

## Abstract

There is little doubt that significant benefits can accrue from carrying out screening mammography of women aged 40–49 in the setting of a highly quality assured service delivery. This will best be achieved using digital mammography to maximise detection rates and trained and high volume reading expert radiologists to apply economic cushions of optimising specificity as well as sensitivity in addition to utilising modern and accurate assessment and tissue sampling techniques that have evolved.

## Article

The National Health Service Breast Screening Programme (NHSBSP) in the UK routinely invites women aged 50–70 years for mammographic screening on a 3 yearly basis. However debates have been underway for many years as to whether or not extending screening to include women age 40–49 should be considered. The age of 50 was chosen as a surrogate for the menopause and because of the rising incidence of breast cancer at this age, but there is little data to support this as the ideal start age for mammographic screening[1]. Post-menopausal breast tissue involutes and mammographic abnormalities are easier to detect than in pre-menopausal breasts, but with women reaching the menopause from their 30's to their 60's, the advent of hormone replacement therapy (HRT) use in post-menopausal women, and with the advent of digital mammography, this feature diminishes and so the argument for commencing screening at 50 is weakened.

In 1997 a meta-analysis of all eight randomised controlled trials (RCTs) which included data on breast screening in women under 50 concluded that there was a statistically significant 18% reduction in breast cancer mortality in women aged 40–49 who were mammographically screened compared to the controls[2]. However, these RCTs were performed from 1963 to 1982, and since then there have been many improvements in technology, such as the introduction of full field digital mammography, and in the expertise of radiologists in interpreting mammograms. It is beyond reasonable doubt therefore that this reduction in mortality of 18% could be substantially improved upon today.

The only randomised study to investigate this issue since the meta-analysis in 1997 is the UK study. This study was set up in 1991 to study the effects of mammography specifically for women aged 40–41 with annual mammograms. Screening in the trial was by two-view mammography at the first screen, with single view thereafter. Interim results published in 2005 show that screening has identified an increase of 8% of invasive cancers, and predicted that deaths at 10 years are 10–11% less than the control group[3]. However, this figure will probably increase with continued follow-up as it is known that the improvement in mortality for younger women screened improves with length of follow up. The meta-analysis mentioned above showed that at 7 years of follow up, there was no mortality reduction for younger women, but at 10.4 years a non-significant improvement of 16% was found, and at 12.4 years, the benefit had risen to statistically significant 18%[2]. Beyond RCTs, and with a specific reference to the 40–49 group, Tabar et al showed that after a 20 year follow up of implementation of service screening in the Swedish Two-Counties, there was a significant 48% reduction in breast cancer mortality for the screening group-and a non-significant 19% reduction in unscreened women[4]. Such results demonstrate the need for high quality, specialisation and a small number of expert centres being involved.

The UK study reported lower than expected detection rates and this can be partly attributed to the use of single-view rather than two-view mammography for incident screens, and the fact that early contributors were not working to the mammographic standards (such as optical density) that we now regard as acceptable and which showed a significant improvement over the second half of the trial period.

In addition to the potential reduction in mortality, screening mammography also leads to benefits in tumour characteristics and eligibility for conservative surgery and less toxic systemic therapy thus leading to a better quality of life[4]. It is known that increasing size and stage of breast cancer is associated with more toxic treatment and poorer cosmetic outcome[5,6]. Studies show that breast cancers diagnosed by screening mammography are of smaller size, at an earlier stage and show more favourable nodal status compared to non-screening detected cancers[7-11]. The Gothenberg breast screening trial showed that the effects of invitation to screening on the incidence of lymph node positive disease closely paralleled the beneficial effects of invitation on breast cancer mortality[12]. With strong evidence that breast conserving surgery is a safe alternative to mastectomy, screen detection of cancer allows women the option of breast conserving surgery and minimally invasive axillary surgery as an alternative to mastectomy and axillary node clearance.

Should mammography be introduced to women under 50, then the current 3 yearly invitation is too infrequent to be effective. While DCIS currently accounts for 20% of all screen-detected cancers, the aggressive cancers that occur in younger women need to be detected early to allow benefits of screening and treatment. A Swedish study showed that the two yearly screening interval was not effective in detecting the more aggressive tumours with poor prognosis. The authors concluded that women under 50 should have mammograms every 12–18 months to gain most benefit from screening[13,14]. Moreover, a recent American study demonstrated that women with breast cancer were diagnosed with DCIS and earlier stage invasive disease more frequently if they had mammography at least annually than if they had mammograms less frequently[7].

X-ray screening programmes are obviously not without risks!. The potentially harmful consequences of mammographic screening include lead time effect, radiation exposure, false positive results and over-diagnosis of breast disease[15]. For a screening programme to be acceptable, the risks must be outweighed by the benefits. Critics of breast screening programmes for younger women argue that the extra radiation exposure is unacceptable for denser, pre-menopausal breasts. However, it has been found that the significant factor for the dose of radiation in screening is not age, but the size of the breast and the quality of the radiological equipment[16]. A study group in 2005 set up a radiation risk model to estimate the number of radiation-induced deaths, and assess the overall balance of lives saved by screening and deaths caused by radiation exposure for women aged 20, 30 and 40. For women under 40, the risks of regular mammograms outweighed the benefit of screening, but for women over 40, annual 2 view mammography was deemed beneficial if screening conveys a 20% mortality improvement. If the perceived reduction in mortality is only 10%, then the risk:benenfit ratio is zero[17]. Therefore, if conclusive evidence can be found that the improvement in mortality from screening is anything above 10% then the radiation risk will be outweighed by the benefits of breast cancer screening for women over 40. However such estimates have been based on the use of film mammography rather than full-field digital mammography which has a higher accuracy and allows a lower radiation dose to be used[18].

Another argument against screening in younger women is the decreased sensitivity of mammography in the younger age group. While the sensitivity of film mammography is poorer for younger women, a recent multi-centre study involving a total of 49,528 asymptomatic women presenting for screening showed that digital mammography was more accurate than film mammography in women under 50, and those with dense, pre- or peri-menopausal breasts[18]. Therefore along with other imaging techniques, such as ultrasound and magnetic resonance imaging, accurate diagnosis of breast cancer can be improved further with modern imaging techniques.

Investigation of suspicious mammographic findings must include a tissue diagnosis to either identify the type of malignancy, or exclude its presence. For screening patients, the lesions are often impalpable, and in previous years, open surgical biopsy was often performed for tissue diagnosis. For false positive mammograms, this meant that women were exposed to unnecessary surgical procedures. However, the use of ultrasound guided and stereotactic core biopsies has meant that most of the false positive results from screening can now be diagnosed with relatively non invasive procedures. A recent meta-analysis and multi-institutional trial showed that stereotactic core biopsy had a similar false negative rate (1–3%) to that of wire guided open biopsy[19-21].

Anxiety associated with recall for suspicious mammograms is a concern for screening groups of all ages. However studies have found that for women with normal or benign results at recall, the distress is short lived and diminishes with time[22]. It was also shown that there was no evidence of long term anxiety or depression for the majority of these women recalled with false positive results[23]. Anxiety is highest in women awaiting surgical biopsy, but with non surgical core biopsies and rapid release of results of investigations, these anxieties should not be long standing for these women. In one study, the majority of women asked said that they were satisfied with the screening programme despite the potential for false positive results[24] and in another study, women recalled for benign disease were almost unanimously content with participating in the breast cancer screening programme[22].

Cost-effectiveness is a key issue in the NHSBSP as the incidence is so much lower in this group and the difficulty of finding trained staff to run yet another extension to the programme might cause it to collapse. The additional workload to the programme from adding this age group would be over 80 % compared to the currently expanded service and approximately 140% more than the original Forrest specification. Nevertheless we are in a situation where cancers in this age group are increasing and breast cancer remains the single commonest cause of death among women aged 35–50 and one third of life years lost are to those women diagnosed in their 40 s. All this at a time of arguably the greatest devastation to family and economic life[25].

International opinion is changing regarding the recommendations for mammography in women under the age of 50. By informing women of the potential harms and benefits of screening, they can make their own choices as to the age at which they start screening programmes. In one study, when pros and cons of screening were explained to women, the majority opted for screening [26]. The American Association of Family Physicians, the Canadian Task Force for Preventive Health, the American Medical Association, the American Cancer Society and the US Preventive Services Task Force all support mammography screening beginning at age 40 with appropriate counselling.

In conclusion, there is little doubt that significant benefits can accrue from carrying out screening mammography of women aged 40–49 in the setting of a high quality assured service delivery. This will be best achieved using digital mammography to maximise detection rates and trained and high volume reading expert radiologists to apply economic cushions of optimising specificity as well as sensitivity in addition to utilising modern and accurate assessment and tissue sampling that have evolved.

## References

[B1] Kopans DB (2005). Informed decision making: age 50 is arbitrary and has no demonstrated influence on breast cancer screening in women. AJR.

[B2] Edwards R, Smith RA, Rutledge JH, Smart CR (1997). Benefit of screening mammography in women aged 40–49: A new meta-analysis of randomised controlled trials. J Natl Cancer Monogr.

[B3] Tabar L, Yen MF, Vitak B, Chen HH, Smith RA, Duffy SW (2003). Mammography service screening and mortality in breast cancer patients: 20-year follow-up before and after introduction of screening. Lancet.

[B4] Moss S, Waller M, Anderson TJ, Cuckle H Randomised controlled trial of mammographic screening in women from age 40: predicted mortality based on surrogate outcome measures. Trial Management Group. Br J Cancer.

[B5] Barth RJ, Gibson GR, Carney PA, Mott LA, Becher RD, Poplack SP (2005). Detection of breast cancer on screening mammography allows patients to be treated with less-toxic therapy. AJR Am J Roentgenol.

[B6] Dewar JA, Benhamou S, Benhamou E, Arriagada R, Petit JY, Fontaine F (1988). Cosmetic results following lumpectomy, axillary dissection and radiotherapy for small breast cancers. Radiother Oncol.

[B7] Taylor ME, Perez CA, Halverson KJ, Kuske RR, Philpott GW, Garcia DM (1995). Factors influencing cosmetic results after conservation therapy for breast cancer. Int J Radiot Oncol Biol Phys.

[B8] Freedman GM, Anderson PR, Goldstein LJ, Hanlon AL, Cianfrocca ME Routine mammography is associated with earlier stage disease and greater eligibility for breast conservation in breast carcinoma patients age 40 years and older. Cancer.

[B9] Spillane AJ, Kennedy CW, Gillett DJ, Carmalt HL, Janu NC, Rickard MT, Donnellan MJ (2001). Screen-detected breast cancer compared to symptomatic presentation: an analysis of surgical treatment and end points of effective mammographic screening. ANZ Journal of Surgery.

[B10] Van der Hage JA, van de Velde CJ Clear-cut beneficial effect of breast cancer screening on the trend in breast-conserving surgery. Nederlands Tijschrift voor Geneeskunde.

[B11] Duffy SW, Tabar L, Fagerberg G, Gad A, Grontoft O, South MC (1991). Breast screening, prognostic factors and survival: results from the Swedish two county study. Br J Cancer.

[B12] Bjurstam N, Bjorneld L, Warwick J, Sala E, Duffy SW, Nystrom L The Gothenburg Breast Screening Trial. Cancer.

[B13] Committee and Collaborators, Falun meeting (1996). Report of the meeting on mammographic screening for breast cancer in women age 40–49. Int J Cancer.

[B14] Tabar L, Vitak B, Chen HH, Prevost TC, Duffy SW (1999). Update of the Swedish Two-County Trial of breast cancer screening: histologic grade-specific and age-specific results. Swiss Surg.

[B15] Jatoi I (1999). Breast Cancer Screening. Amer Jour of Surg.

[B16] Young KC (2002). Radiation doses in the UK trial of breast screening in women aged 40–48 years. British Jour of Radiol.

[B17] Berrington de Gonzalez A, Reeves G Mammographic screening before age 50 years in the UK: comparison of the radiation risks with the mortality benefits. Br J Cancer.

[B18] Pisano E, Gatsonia C, Hendrick E, Yaffe M, Baum J Diagnostic performance of digital versus film mammography for breast-cancer screening. NEJM.

[B19] White RR, Halperin TJ, Olson JA (2001). Impact of core-needle breast biopsy on the surgical management of mammographic abnormalities. Ann Surg.

[B20] Verkooijen HM, Peeters PHM, Buskens E (2000). Diagnostic accuracy of large-core needle biopsy for non palpable breast disease: a meta-analysis. Br J Cancer.

[B21] Brenner RJ, Bassett LW, Fajardo LL (2001). Stereotactic core-needle breast biopsy: a multi-institutional prospective trial. Radiology.

[B22] Ekeberg O, Skjauff H, Karesen R (2001). Screening for breast cancer is associated with a low degree of psychological distress. Breast.

[B23] Lampie C, Thurfjell E, Bergh J, Sjoden PO (2001). Short- and long-term anxiety and depression in women recalled after breast cancer screening. Eur J Cancer.

[B24] Schwartz LM, Woloshin S, Sox HC, Fischhoff B, Welch HG (2000). US women's attitudes to false positive mammography results and detection of ductal carcinoma in situ: cross sectional survey. BMJ.

[B25] Yarbrough SS, Braden CJ (2001). Utility of health belief model as a guide for explaining or predicting breast cancer screening behaviours. J Adv Nurs.

[B26] Mokbel K, Lirosi F, al-Sarakbi W, Leris C (2001). Women's views on the introduction of annual screening mammography to those aged 40–49 years (a pilot study). Curr Med Res Opin.

